# Assistant Nurses’ Experiences of Life Story Documentation: Insights Into the Construction and Application in Dementia Care Practice

**DOI:** 10.1177/14713012251389470

**Published:** 2025-10-14

**Authors:** Catharina Melander, Karin Zingmark, Therese Lindh, Ingela Jobe

**Affiliations:** 1 Division of Nursing and Biomedical Engineering, Department of Health, Education and Technology, 5185 Luleå University of Technology, Luleå, Sweden; 2 Department of Caring and Ethics, Faculty of Health Sciences, 56627 University of Stavanger, Stavanger, Norway

**Keywords:** assistant nurses, dementia, focus group interviews, life story documentation, nursing, nursing home, person-centered care, qualitative content analysis

## Abstract

Understanding a person’s life story can transform dementia care by promoting a person-centered approach. Documented life stories help staff to personalize care, but further research is needed to understand their construction and usage in care practice. The aim of this study was to explore assistant nurses’ experiences of life story documentation and their practical applications in dementia care practice. Five semi-structured focus groups were conducted, and data were analyzed using qualitative content analysis. The findings consisted of five themes: “Ensuring timing and accessibility when constructing a documented life story,” “Recognizing template limitations in capturing a comprehensive view of the person,” “Refining life story quality and understanding through ongoing dialogues,” “Building connection and support through insights from documented life stories,” and “Harmonizing life histories with evolving care needs in practice.” Our findings highlight the importance of a dynamic and inclusive approach to life story documentation that goes beyond rigid templates. Accessible and well-documented life stories can empower nursing staff to provide responsive and personalized care, enhancing the dignity and quality of life of people living with dementia.

## Introduction

Cognitive impairments such as dementia influence nursing staff’s perceptions of nursing home residents’ personhood (Eritz et al., 2016), making it challenging for staff to form meaningful connections with inhabitants (Qu et al., 2019). As the disease progresses, those with dementia may encounter difficulties in verbally expressing their needs, emotions, and preferences in order for others to understand them (cf. Nguyen et al., 2022). As life stories may be understood as narratives through which identity, experiences, and meaning are expressed, a deep understanding of them is central to preserving identity (Kitwood, 1997a) and fostering recognition and understanding (Baxter et al., 2020). In this way, life stories provide an important foundation for person-centered care (Cooney & O’Shea, 2019; Ejaz et al., 2022), which improves well-being in persons with dementia residing in nursing homes (Ho et al., 2021) and is vital for providing high-quality dementia care (cf. Lee et al., 2022; McCance et al., 2011).

Narrative care has been described in various ways, but all definitions seem to share the common essence of placing storytelling at the very center of care practices (Villar & Westerhof, 2023). Bamberg (2006, pp. 63-64) divides the concept of narratives into big stories and small stories. Big stories are often elicited in interview settings for research or therapeutic purposes, where narrated events and episodes are woven into a coherent life story. In contrast, small stories refer to narratives shared in everyday interactions, often about ordinary events in daily life. According to Villar and Westerhof (2023), care practices in which small stories are a natural part may promote personhood and narrative agency in people living with dementia. They also present three strategies to establish and maintain small stories in institutional care: (1) prompting and sustaining narratives when they appear; (2) recognizing and valuing non-verbal cues and embodied narratives; and (3) promoting narrative environments. The use of small stories underlines the importance of narratives in daily care practices, and this perspective can be further extended to the use of documented life stories.

Integrating a person’s documented life story into caregiving enhances the person-centered approach by fostering strong connections between nursing staff and those they support (Dellkvist et al., 2024; Doran et al., 2019) and provides care that reflects the person’s unique needs and expectations (Hedman et al., 2022; Ho et al., 2021). Such integration entails knowledge and understanding of the person’s values and preferences, leading to care that can be adapted to the individual (cf. Ejaz et al., 2022). It further enhances empathy and strengthens relationships with persons with dementia (Doran et al., 2019; Muller et al., 2022; Qu et al., 2019) creating confidence and fulfillment in nurses toward their roles as caregivers and reducing their workload burden (Muller et al., 2022; Qu et al., 2019). Using documented life stories in care practice may also affect the resident’s well-being by strengthening identity and social interactions (cf. Kindell et al., 2019), reducing depressive symptoms (Chan et al., 2013), lessening behavioral symptoms, such as agitation and aggression, and improving their quality of life (Eritz et al., 2016). Relatives of persons with dementia find documenting a life story a helpful way to cope with grief and stress (Andersson et al., 2019), and nursing home residents often have positive experiences creating these (Ejaz et al., 2022).

Life story work is a broad therapeutic approach that captures a person’s life experiences through various forms of narrative, such as storytelling, structured interviews, collages, or videos. Life story books are a popular tool in this process, compiling personal memories, photos, and significant life events (Subramaniam et al., 2023). In care practice, life story templates are commonly used to capture a person’s life through photos and chronologically arranged captions. Typically, relatives or nursing staff create, store, and access the templates, with only minimal involvement or control from the person with dementia (Kindell et al., 2014). However, picturing a person’s previous life in a fixed life story template and understanding how the information will be used in care practice may present challenges. Documenting a meaningful life story requires time and collaboration among family members and significant others (Andersson et al., 2019). McKeown et al. (2015) show that assessing nursing staff’s educational needs and providing training in documenting life stories is important, as is clarifying the aims and purpose of the life story (McKeown et al., 2015). Although research shows positive effects of using documented life stories, relying on standardized life story templates in dementia care poses several challenges, for example overlooking individual differences. A life story template may fail to capture the richness of a person’s lived experiences, especially when applied in care settings. Moreover, there is limited guidance on how to creatively and flexibly document and utilize the life story (cf. Berendonk & Caine, 2017, 2019; Gridley et al., 2020). Hence, life story production may be prioritized over practical application (Kindell et al., 2014).

Life story as a tool to capture the unique experiences, memories, and preferences of individuals with dementia, has gained attention in recent years. Despite the recognized importance of life stories in dementia care, there are still gaps in the literature concerning the experiences of such documentation and its usability in care practice. Assistant nurses play an essential role in delivering care and establishing relationships with persons with dementia, often being the ones who spend the most time with them in the nursing home context. This positions them in a central role of gathering a documented life story of the person with dementia, as well as making use of it in care practices. Life stories not only enhances the understanding of each patient as a unique person but can also foster a more empathetic and individualized approach to care. However, little is known about how assistant nurses, who are often at the forefront of direct care, perceive and utilize this method in practice. This study seeks to explore their experiences with life story documentation in dementia care, shedding light on its potential benefits, challenges, and practical applications.

## Aim

The aim of this study was to explore assistant nurses’ experiences of life story documentation and its practical application in dementia care practices.

## Material and Methods

### Design

This study employs a qualitative approach to explore assistant nurses’ experiences of life story documentation and its practical application in dementia care practice. Focus group interviews contributed to a narrative approach to target richness in data. Data were analyzed with a qualitative content analysis method in accordance with Graneheim and Lundman (2004).

### Participants

The recruitment process followed purposive sampling. Operation managers from two municipalities, were provided with detailed information about the study and asked for their consent to proceed. Once approval was obtained, each manager purposefully selected nursing homes within their municipality that specialized in dementia care and actively integrated life stories into their practices (see Appendix 1 for an example). Nursing home managers were then informed about the study and asked to distribute study details and consent forms to the assistant nurses. The inclusion criteria required at least one year of experience working with persons with dementia and prior experience as a contact person. (In Sweden, a contact person is a designated staff member responsible for coordinating and personalizing the care of an older person, acting as a primary point of contact for both the resident and their family). Those interested in participating contacted either their manager or one of the researchers to arrange interviews. All individuals who expressed interest in participating were included, resulting in 16 female assistant nurses from three nursing homes. The participants’ ages ranged from 31 to 63 years (mean = 53), and their care work experience spanned 3 to 40 years (mean = 29). Additionally, 14 of the participants had some form of specialized training in the care of older adults and/or persons with dementia. All three nursing homes were publicly run (municipal) facilities. Upon admission, relatives were given the LS template, and when it was returned, the assistant nurses strived to use it as a tool to support person-centered care.

### Data Collection

The first author (CM), who had prior experience in conducting qualitative interviews and focus groups conducted five focus groups between February and March 2022, along with the third author (TL). Each group consisted of three or four participants, and the interviews were conducted during the participants’ working hours at their respective workplaces. This method was chosen to encourage participants to share, discuss, and refine their views and opinions within a group setting, fostering deeper insights through interaction (cf. Morgan, 1997).

Interviews took place using a semi-structured interview guide with open-ended questions. Key focus areas included prompts such as: “Can you describe how you use life stories in your daily work with individuals with dementia?” and “How does the life story influence your work?” Follow-up questions included: “How do you feel about that?” “Can you elaborate?” and “How does knowing this information help you?”

Data were collected through digital audio recordings, and lasted between 30 and 45 minutes, with an average duration of 36 minutes. Data collection continued until no new experiences or perspectives emerged in the focus groups, which indicated data saturation. The third author transcribed the recordings verbatim shortly after the interviews, and each participant was assigned a unique code number during transcription. The first author conducted the initial coding, and emerging categories were continuously discussed and refined within the research team to reach the most coherent and reasonable interpretation.

### Data Analysis

The first (CM) and third (TL) authors analyzed the data, following the approach outlined by Graneheim and Lundman (2004). The transcribed interviews were read repeatedly to gain a comprehensive understanding of the data. Meaning units were then identified, translated into English, and condensed while preserving their core meaning. These condensed units were coded based on their content.

Coded content was then grouped based on similarities, leading to the creation of subcategories. By working closely with the text during the coding process, we ensured that these subcategories reflected the nuances of the data. Through ongoing reflections and discussions within the research group, subcategories were merged into higher-order categories. An interpretive process led to the development of overarching themes that emerged from the data, capturing deeper meanings and patterns within the participants’ responses. These themes were derived through a careful analysis of the data, ensuring that they reflected the key insights and nuances present in the participants’ experiences. This iterative process continued until the themes were exhaustive and mutually exclusive, ensuring that no further abstraction was deemed appropriate.

### Ethical Considerations

This study was approved by the Swedish Ethical Review Authority (Dnr. 2020-07041). Before the interviews, potential participants were provided with detailed information about the study’s focus, confidentiality, and their right to withdraw at any time. Verbal and written consent were then obtained from all participants.

Given that the study explores potentially sensitive values and views, care was taken to ensure that participants were not exposed or identifiable based on their thoughts or opinions related to their workplace. To safeguard confidentiality, data were carefully managed and protected using established data security principles, with access restricted to authorized members of the research team.

## Findings

The findings consisted of five themes, presented in Table 1. These are followed by descriptive texts and illustrative quotes.

**Table 1.** Overview of Themes

Theme
Ensuring timing and accessibility when constructing a documented life story
Recognizing template limitations in capturing a comprehensive view of the person
Refining life story quality and understanding through ongoing dialogues
Building connection and support through insights from documented life stories
Harmonizing life histories with evolving care needs in practice

### Ensuring Timing and Accessibility when Constructing a Documented Life Story

The process of constructing a documented life story involved a relationship between the timing of its introduction and its potential effectiveness in care. The process was typically initiated by providing relatives with a life story template soon after the person with dementia relocated to the nursing home; subsequently, assistant nurses would collect the completed template. When introduced, the life story documentation risked becoming a burdensome task for already overwhelmed and exhausted relatives. In this context, the construction of a documented life story could be perceived by many as yet another administrative task, rather than a meaningful opportunity to influence the quality of care their loved one would receive. If approached later, relatives could better reflect on and share their loved one’s life story. Assistant nurses also highlighted the importance of relational closeness between the person and the relatives constructing the documented life story to include more detailed and current insights. They emphasized that relatives might not have access to all parts of the person’s life, for example, aspects that were kept secret out of shame or personal integrity. As described by one assistant nurse, “We all have secrets.”

Assistant nurses typically encouraged collaboration between relatives and the person with dementia when constructing the documented life story. However, the memory-dependent nature of the questions in the life story template were often challenging for the person. Although it was time-consuming for assistant nurses to construct the documented life story directly with the person, a deeper narrative could emerge. The success of this approach required timing, patience, and the creation of positive, relaxed moments when the documentation could take place.

When reflecting on the construction of documented life story, assistant nurses noticed variations in the way they were documented and used in different nursing homes. Recognizing the need for a uniform approach, they advocated the adoption of a standardized template for the design of an life story. A universally applied system would ensure that the life stories were accessible to all caregivers in different care settings. Assistant nurses viewed the documented life story as a dynamic tool that should evolve even after its initial construction. However, updates were rare, and the documented life story often stagnated. To construct a relevant and accessible life story, they identified two key strategies: maintaining easy access to the documented life story and actively encouraging staff to update it regularly. Unlike other documentation processes, the roles and responsibilities for updating the documented life story were perceived as ambiguous. Among other circumstances, there were no clear guidelines on how it should be used and stored.

Life stories can be found everywhere. Some have it in the walker, in the basket, under all the stuff, and sometimes the life story is in a binder in the staff office. But sometimes it can be in someone’s residence. And yes, there would be some kind of structure, and you would have it somewhere, so it is easily accessible instead of just locking it up. (Assistant Nurse 1, Focus Group 5)

To strengthen the construction and continuity of documented life stories, assistant nurses suggested implementing digital solutions, such as laptops or tablets. A digital format would streamline updates and improve accessibility. Furthermore, they recommended that all important information relating to the person, including the documented life story, should be stored in the same format and in the same place. This would ensure that the most up-to-date information would be readily available and improve accessibility, timely updates, and create a comprehensive picture of the person’s life and care, ultimately ensuring that the documented life story remained dynamic.

### Recognizing Template Limitations in Capturing a Comprehensive View of the Person

According to assistant nurses, the life story template frequently failed to capture the essence of the person with dementia, which posed significant challenges when striving to utilize it in daily care. The template typically included captions where family members provided information about the person’s preferences regarding food, personal hygiene, and the use of tobacco or alcohol. It also covered various aspects of the individual’s life, such as their childhood, middle age, living arrangements, family life, personality, relationships, occupation, religious beliefs, and significant life events. The documented life story was intended to provide a comprehensive picture of the person; however, it was largely retrospective and superficial, providing little insight into current preferences or daily habits, such as the person’s clothing or drink preferences. Assistant nurses found the life story template to be limited, often containing yes or no questions that did not provide meaningful and rich descriptions of the person’s interests, habits, or personality traits - important factors that would help them understand and meet individual needs. For example, important details about whether a person preferred mornings or evenings and about the person’s drinking habits or financial behavior were often missing, leaving nurses unsure how to provide truly person-centered care. This lack of detailed and nuanced information forced them to constantly interpret and “fill in the gaps,” a process they found challenging. In one case, checkboxes in the life story template indicated an interest in “mushroom picking,” which did not provide any real understanding of the person’s involvement - whether it was a love of nature, a passion for cooking, or a social activity shared with family. This vagueness left nurses unsure how to integrate such information into care plans.

Parts of the life story are not very descriptive and need to be interpreted. Alcohol habits: ‘Yes’. Is this an alcoholic? Someone who drinks a glass of wine every other Sunday or what are these alcohol habits? It requires interpretation. Free for more investigations... (Assistant Nurse 2, Focus Group 1)

In addition, important life events, such as marriages or careers, were also superficially documented in the life story template. There was little or no information about emotional responses to these experiences, such as whether the person had been happy in their marriage or found satisfaction in their career. This further limited the depth of understanding that assistant nurses gained from the documented life story. They emphasized the need for a more thorough and detailed approach to documenting life stories, as the existing template, which often relied on short answers, failed to capture the complexity of the person’s life and preferences. They highlighted the need for more detailed free-text entries to ensure that the person’s life was truly reflected. According to assistant nurses, a meaningful documented life story should be crafted like a story, rather than a random collection of experiences stacked without intention. In essence, the documented life story often fell short of its intended purpose and lacked the depth required to truly understand and support the person with dementia.

In their reflections, assistant nurses highlighted a significant gap in documented life stories in that they often omitted difficult or traumatic events, even though it was often requested in the life story template. This meant that the documented life story did not fully capture the individual’s life, especially as many persons with dementia may relive traumatic moments from their past. Without such insight, they may miss important aspects of the person’s emotional and psychological needs. By incorporating these traumas into the documented life story, assistant nurses were able to better understand and validate the individual’s experiences, which not only helped create a sense of safety but also enabled more compassionate care. They shared a striking example how understanding a person’s past experiences could provide crucial context for their present behaviors:

He would unscrew all the light bulbs... everywhere. He panicked and pulled out all the wires. Finally, an acquaintance came to see him, and he laughs and says: ‘I see he’s pulling out a lot of lights here.’ ‘Yes,’ I say, ‘I wonder what that is...’ ‘Well, hasn’t anyone told you that he almost burned to death as a child?’ Then, you see…it would have been good to know. (Assistant Nurse 3, Focus Group 3)

### Refining Life Story Quality and Understanding through Ongoing Dialogues

When using the documented life story in dementia care, assistant nurses acknowledged themselves as a third party, seeking to absorb and understand the information in the life story. As these could vary considerably in the amount of information and were rarely written in a way that could be directly applied in daily care, communication was important. Refining the quality of the life story through an ongoing dialogue with the person with dementia and with relatives was essential to fill in informational gaps, comprehend the information, and use it in daily support and care.

Yes, that’s how it is sometimes, and then it’s not so descriptive [the information in the documented life story ---]. So, you get to interpret. I usually take a pad and pen with me actually and ask my own questions [to relatives] because I want to know things like that. (Assistant Nurse 2, Focus Group 1)

When there were gaps in the life story, assistant nurses were proactive, actively seeking additional information from the person with dementia and from relatives. Through both verbal and non-verbal interactions, they engaged with the person asking questions or interpreting body language to gain insights from the documented life story in a way that could enhance care. Assistant nurses emphasized that relatives often would provide additional details if asked and explain documented information. This refinement entailed that information was understood correctly, used as intended, and could be applied in daily support and care. Through these dialogues, assistant nurses also gained valuable insights into recent changes in the person’s interests, preferences, and behaviors. They could then adopt support in ways that ensured that care remained personalized and responsive to the needs of the person with dementia. This continuous exchange of information with relatives not only enriched the information in the documented life story and improved its quality but also ensured that care would be more precisely tailored to the individual.

### Building Connection and Support through Insights from Documented Life Stories

Increased knowledge gained through the documented life story was fundamental for assistant nurses in creating understanding and meaningful personal connections with the nursing home resident. A well-documented life story provided them with an important starting point for understanding the person’s past and identity. Reading the life story offered valuable insights that enabled them to create a more holistic understanding of the person. However, assistant nurses had time constraints and found it difficult to retain information from multiple life stories and fully remember details of each person’s life. The documented life story played a particularly important role at the beginning of the relationship between the assistant nurse and the person with dementia, helping nurses to understand the individual’s history and recognize patterns of behavior, such as anxiety or changes in mood. Creating a sense of security and comfort for the person with dementia required this prior knowledge.

[The life story support] By getting to know each other and if you know a lot about each other, that’s security. That the person knows ... that she knows, she knows this about me. (Assistant Nurse 3, Focus Group 2)

The documented life story served as a rich source of conversation starters in everyday interactions, including basic details such as travel history, occupation, previous residences, and family connections. Although simple, these details helped to create a sense of belonging and trust between the assistant nurse and the person with dementia. Familiar topics, such as place of birth and previous occupations, which often remained in the person’s memory, were invaluable in creating meaningful conversations and support care. Assistant nurses found that these personal details enabled them to respond more effectively when they mentioned, for example, the names of important people from their past.

She often asks if the children are dead in the evenings after 4 p.m. Her first child died when he was four years old; it feels very important that the children are alive. So, it’s great to know all their names. (Assistant Nurse 1, Focus Group 4)

By using information from the documented life story, assistant nurses were able to create moments of recognition and comfort, as they could stimulate memory and reinforce the person’s identity. Significant life events documented in the life story were particularly useful in evoking memories, supporting meaningful interactions, and promoting a positive emotional state. Ultimately, this approach deepened the connection between assistant nurses and those with dementia, improving the quality of care and the person’s overall well-being.

### Harmonizing Life Histories with Evolving Care Needs in Practice

Integrating information from the documented life story into the daily support and care required a flexible and dynamic approach that harmonized life histories with evolving care needs. Assistant nurses highlighted the importance of adapting the life story information to align with the current circumstances and condition of the person with dementia. Instead of strictly applying details from the documented life story, they emphasized the need to interpret and adapt this knowledge in real time to reflect the person’s current state and ensure that support and care remained both relevant and responsive. For example, documented habits from the past, such as standing up in the shower, may pose a safety risk today due to changes in mobility or health, requiring them to consider how such preferences could be safely integrated into daily care. Another example highlighting the importance of aligning care with expressed preferences of persons with dementia, where discrepancies could lead to discomfort or distress, was the following:

In her life story, she has three boys, and they have ticked that she wants the room darkened. But she’s afraid of the dark, very afraid of the dark. She says herself that she doesn’t want the room darkened at all. (Assistant Nurse 2, Focus Group 4)

Moreover, assistant nurses acknowledged that the documented life story information sometimes conflicted with real-time observations. For example, a person documented as antisocial could show social tendencies in the nursing home environment, engaging with both staff and other residents. This discrepancy highlighted the need to recognize that past behaviors may change over time, as preferences, abilities, and interactions could change. While the documented life story was a valuable tool to tailor activities to the person’s interests and previous occupations, assistant nurses acknowledged that these preferences might evolve, especially as persons adapt to new settings like nursing homes. Activities once central to a person’s identity, such as household chores or crafts, might lose their appeal, prompting assistant nurses to offer alternatives better aligned with the person’s current physical and emotional state. For example, if a person had previously preferred outdoor activities but can no longer manage long walks, assistant nurses might instead offer shorter walks or time on a balcony. Still, honoring the person’s life history and identity remained a priority, as long it was appropriate and safe.

Using the documented life story in care required a continuous assessment to harmonize with the person’s current needs and respond to shifting conditions and wishes. Assistant nurses remained open to support new and emerging wishes, thus respecting the person’s autonomy and current sense of self. By harmonizing the documented life story, as a reflection of the past and the reality of the individual’s present state, they delivered support and care that was both grounded in the person’s life history and sensitive to changing needs, ensuring that daily life and care were personalized and meaningful. A striking example was a person with dementia expressing a long-suppressed desire to sing, not previously visible in his personality or interests.

A man who had never sung. He sang the whole conversation and others heard it. He sang and sang. And I asked, ‘Have you been singing all your life?’ No, that was when he told me when he went to school. ‘We weren’t allowed to sing at school,’ because he sang ‘rather than well,’ said the schoolmistress. Then he wasn’t allowed to join in, because he sang so badly. So when he got older, he wanted to sing, and when he got dementia, then he sang... (Assistant Nurse 2, Focus Group 5)

## Discussion

This study aimed to explore assistant nurses’ experiences of life story documentation and its practical application in dementia care practice. It was evident that the documented life story was an important tool, and the results clearly elucidate the essential aspect of moving beyond the life story template. By integrating depth, dialogue, and flexibility when striving to make practical use of the documented life story, assistant nurses could utilize it for personalized care. Assistant nurses endeavored to move beyond surface-level data in life story documentation, integrating personal nuances, and capturing meaningful details that provided insight into the person’s identity, preferences, and values. Recognizing the complexity of each person’s life story and personal history is crucial to personalizing care in a way that respects each person’s individuality. These are important aspects to consider when constructing and using documented life stories to inform support and care.

Our findings indicate that template-based life story documentation caused limitations in gaining a comprehensive view of the person. This aligns with Möllergren and Harnett (2024), who demonstrated that using such templates can yield two distinct narratives: one depicting the person as a patient with dementia and another portraying the person as they were before symptoms emerged. Furthermore, these templates can unintentionally create a static, one-dimensional view of the person, sometimes suggesting an overly simplified or idealized life without challenges. The structure of the life story template critically influences the type and depth of information collected; essentially, the questions posed determine the responses received. Strategies for allowing more flexible, adaptive formats that accommodate personalized information are important (Harrison Dening, 2023). Participants in our study, as well as in Dellkvist et al. (2024), highlighted the use of digital life stories to improve accessibility, timely updates, and clarification of life story information. To have the life story accessible may foster person-centred care since it ensures that nursing staff can quickly understand and respond to the individual person’s needs.

It is paramount to involve the person with dementia as much as possible in the documentation process to ensure that their life story truly reflects their distinct personality, wishes and preferences (Harrison Dening, 2023). Our findings indicate limitations in the template in promoting participation of the person to document their own life story, which highlights the need to consider how structured approaches can either enable or constrain meaningful participation. As shown by Kłosińska and Leszko (2023), people can create complex narrations about themselves despite severe cognitive difficulties. To promote participation in life story documentation, it is important to create an environment where people can speak freely, allow for pauses when needed, encourage familiar images and approach each topic sensitively. Additionally, utilizing different formats to accommodate different persons may be helpful, such as scrapbooks, photo albums, auditory life stories, memory boxes, and illustrated life spans/family trees. Still, the structure of the template can significantly affect how well people with dementia are able to engage in and contribute to the creation of their documented life story (Harrison Dening, 2023).

Shared narrative structures may provide a useful framework for life story documentation, helping persons with dementia actively participate in the process. For example, by organizing life stories around familiar patterns—broad life stages, such as childhood or their first marriage; recurring events, like family dinners; and significant moments, such as the death of a loved one or a memorable vacation—nursing staff can create a more accessible and meaningful storytelling experience. Further, identifying meaningful locations tied to family events may be more accessible than recalling specific dates (McAdams, 2021). Establishing connections between events and linking life stages to specific activities may provide coherence (Kłosińska & Leszko, 2023). Including aspects such as professional experiences in the life story, not just to remember past jobs but also to understand current roles (Kłosińska & Ziółkowska, 2023), regional identity, emotional closeness in relationships, and the broader social context of family history can enhance the meaning of personal experiences. This integration helps shape the person’s current identities and values, demonstrating that life story documentation can serve as more than a record of the past—it can actively support a person’s evolving sense of self (Kłosińska & Leszko, 2023). Results from other studies, together with our findings, indicate that having an inclusive template and a flexible approach when gathering information and documenting the life story is essential not only to create a comprehensive view of the person, but also to facilitate the person with dementia to produce their own life story. To have a life story that the person can remember and share has the potential to promote their self-esteem and identity (cf. Kitwood, 1997a; 1997b).

Our findings showed that applying life story information to real-time situations implied a need to harmonize past and present; it illuminates the balancing act of respecting the person’s identity amid changing abilities. It is necessary to continuously reassess life story information to ensure it aligns with the person’s health and lifestyle needs and makes certain that care remains relevant and responsive (cf. Brown et al., 2020; Gallagher-Thompson et al., 2020). Emphasizing individuality involves preserving connection to the past, while also fostering an environment that supports both uniqueness and personal growth (Möllergren & Harnett, 2024). Given our findings, we suggest the need for specialized training for nursing staff to effectively document, interpret, and apply life story information, emphasizing the skills required to manage this information with depth, sensitivity, and flexibility. Additionally, it is important to establish clear guidelines to support the construction of the life story, determining who is responsible for each step and how updates should be conducted. As concluded by Möllergren & Harnett (2024), it is also crucial to address the challenges of creating a life story, such as the risk of oversimplifying a person’s identity and reducing their experiences to fixed narratives. To truly reflect a person’s evolutionary journey, life story templates must remain flexible, inclusive, and adaptable to changing perspectives and personal growth. Our findings highlight the necessity to move beyond the life story template, integrating depth, dialogue, and flexibility in documentation, which provides the potential to enable people with genuine opportunities to be and to do things according to one’s personal life values, hence supporting a dignified life (cf. Nussbaum, 2011). Through this approach, life story documentation becomes a tool not only for recording past information but for actively supporting each resident’s current and potential capabilities, promoting a life of value and meaning despite limitations.

## Methodological Strengths and Limitations

This study involved five focus groups with a total of 16 participants, all of whom were female assistant nurses. The participants were knowledgeable, drawing on many years of professional experience in dementia care. Their insights provided a solid foundation for exploring life story documentation and its practical application. Transferability is supported by a clear description of the sample, data collection, and analytic procedures. Data was collected from three different nursing homes across two municipalities, striving to build variation of contexts and increase the potential for transferability to similar settings. To ensure consistency, the first and third authors participated in all focus group interviews. The third author transcribed the material, and the first author conducted the initial data analysis. Throughout the analysis, we continuously returned to the transcriptions to minimize risk of misinterpretation. Credibility was strengthened through ongoing discussions within the research group, iterative refinement of codes and themes, and the use of illustrative quotations from participants (cf. Graneheim & Lundman, 2004).

Despite these strengths, some limitations should be noted. While the sample size was sufficient to identify recurring patterns and highlight variations, it is possible that certain experiences or perspectives were not captured. The homogeneity of the sample, limited to one gender and profession, may have constrained the diversity of viewpoints. Additionally, although data were gathered from different nursing homes, the findings remain context-specific and may not be readily transferable to other healthcare systems, regions, or professional roles. Cultural, institutional, and organizational factors likely influenced participants’ experiences and should be taken into account when considering the applicability of the results in other settings. Future research could benefit from a larger and more heterogeneous sample, including participants from different professional backgrounds and genders, to enhance the breadth and richness of the data.

## Conclusion

In conclusion, our findings highlight the importance of a dynamic and inclusive approach to life story documentation that goes beyond rigid templates. By integrating depth, dialogue, and flexibility, life story documentation can serve as a meaningful tool to preserve identity, promote autonomy, and enhance person-centered dementia care. Ensuring accessibility, acknowledging limitations in templates, and emphasizing ongoing dialogue contribute to a more comprehensive and personalized understanding of each individual. Involving people with dementia in the documentation process is crucial to ensure that their life story accurately reflects their experiences, values, and preferences. Ultimately, accessible and well-documented life stories can empower nursing staff to provide responsive and personalized care, enhancing the dignity and quality of life of people living with dementia.
